# A LONGEVITY PROMOTING FACTOR THAT SUPPRESSES IMMUNITY AND HEALTHSPAN

**DOI:** 10.1093/geroni/igz038.2827

**Published:** 2019-11-08

**Authors:** Arjumand Ghazi, Francis Amrit, Nikki Naim, Ramesh Ratnappan, Julia Loose, Guoqiang Wang, Monica Driscoll, Judith Yanowitz

**Affiliations:** 1 University of Pittsburgh School of Medicine, Pittsburgh, Pennsylvania, United States; 2 Rutgers University, Piscataway, New Jersey, United States

## Abstract

A positive correlation exists between stress resistance and longevity, but emerging evidence suggests that lifespan and stress endurance are physiologically distinct. A major challenge in aging biology has been identifying factors that play distinct roles in these closely coupled processes because genes that promote longevity often enhance stress resistance. Here, we demonstrate that TCER-1, the Caenorhabditis elegans homolog of the human transcription elongation and splicing factor, TCERG1, has discrete and opposite effects on lifespan and stress resistance. We previously identified tcer-1 as a gene that promotes longevity in germline-less C. elegans and reproductive fitness in wild-type animals. Surprisingly, tcer-1 mutants exhibited exceptional resistance against multiple biotic and abiotic stressors, including infection by the human opportunistic pathogen Pseudomonas aeruginosa. Conversely, TCER-1 overexpression increased susceptibility to infection. TCER-1 acted cell non-autonomously to both enhance longevity and repress immunity. Interestingly, TCER-1 inhibited immunity only during the fertile stages of life and not in post-reproductive adults. Elevating its levels ameliorated the fertility loss that follows infection, suggesting that TCER-1 may repress immunity to augment fecundity. Mechanistically, TCER-1 acts through the inhibition of the conserved kinase, PMK-1, as well as through repression of PMK-1-independent, novel antibacterial factors critical for innate immunity. Overall, our data establish key roles for TCER-1 in coordinating immunity, longevity and fertility, and reveal the molecular mechanisms that distinguish length of life from functional aspects of aging.

